# Convergence of multidrug resistance with biofilm formation and hypermucoviscosity in *Klebsiella pneumoniae* from tertiary-care hospitals in Northwestern Pakistan

**DOI:** 10.1017/ash.2026.10405

**Published:** 2026-05-13

**Authors:** Aiman Waheed, Taj Ali Khan, Sajjad Ahmad, Fahad Ahmed, Zahoor Khan

**Affiliations:** 1 Center of Biotechnology and Microbiology, University of Peshawar, Peshawar, Pakistan; 2 Institute of Pathology and Diagnostic Medicines (IP&DM), Khyber Medical University, Peshawar, Pakistan; 3 Public Health Reference Laboratory, Khyber Medical University, Peshawar, Pakistan; 4 Nutrition Innovation Centre for Food and Health (NICHE), School of Biomedical Sciences, Ulster University, Coleraine, N. Ireland, UK

## Abstract

This multicenter study describes the convergence of multidrug resistance, biofilm formation, and hypermucoviscosity in *Klebsiella pneumoniae* clinical isolates from tertiary-care hospitals in Pakistan. The high prevalence of these phenotypes highlights significant therapeutic challenges and underscores the need for strengthened surveillance, infection control, and antimicrobial stewardship.

## Introduction

*Klebsiella pneumoniae* is a Gram-negative member of the Enterobacterales and a major cause of healthcare-associated and community-acquired infections, including pneumonia, bloodstream infections, urinary tract infections, and device-associated infections.^1^ Its clinical significance has increased globally due to the rapid emergence of multidrug-resistant (MDR) and carbapenem-resistant strains, largely mediated by mobile genetic elements encoding β-lactamases.^2^

In addition to antimicrobial resistance, virulence-associated phenotypes of *K. pneumoniae* such as biofilm formation and hypermucoviscosity contribute to persistence, immune evasion, and infection chronicity, particularly in device-associated settings. These traits may complicate treatment outcomes but are often evaluated independently of resistance patterns.^3^ In Pakistan, available data primarily focus on antimicrobial susceptibility patterns of *K. pneumoniae*, with limited integration of phenotypic virulence characteristics.^4^ This study therefore aimed to characterize antimicrobial resistance patterns and selected virulence-associated phenotypes among *K. pneumoniae* isolates from major tertiary care hospitals in Peshawar that serve as major referral centers for all nearby districts of Khyber Pakhtunkhwa (KP), thereby providing region-specific data from a high-burden setting.

## Methods

This multicenter cross-sectional study was conducted from August 2024 to July 2025 across three tertiary care hospitals, namely Khyber Teaching Hospital, Lady Reading Hospital, and Hayatabad Medical Complex in Peshawar, Pakistan, following ethical approval and informed consent procedures. A total of 3,286 clinical specimens (urine, blood, respiratory, wound-associated, and sterile body fluids) were processed using standard microbiological techniques, yielding 248 confirmed *K. pneumoniae* isolates from 953 culture-positive samples.^4^

Species identification was performed using API 20E identification system (bioMérieux, Lyon, France).^4^ Antimicrobial susceptibility testing was conducted using the Kirby–Bauer disk diffusion method, with colistin susceptibility assessed by broth microdilution.^5^ Results were interpreted according to Clinical and Laboratory Standards Institute (CLSI M100, 34^th^ edition) criteria. MDR and extensively drug-resistant (XDR) phenotypes were defined using ECDC/CDC criteria.^6^

Hypermucoviscosity was assessed using the string test, and biofilm formation was quantified using a microtiter plate assay with optical density-based classification.^7^ Whole-genome sequencing and molecular typing were not performed; therefore, genetic determinants and clonal relatedness could not be assessed.

## Results

Among 3,286 clinical specimens, 29.0% yielded bacterial growth, of which 68.3% were Gram-negative. *K. pneumoniae* accounted for 38.1% of Gram-negative isolates (n = 248). This relatively high proportion should be interpreted cautiously, as differences in diagnostic practices, sampling strategies, and patient case-mix may limit generalizability.

Isolates were evenly distributed across participating hospitals (∼32%–34% per center), suggesting no clear institutional clustering. Urine was the most frequent source of isolation (33.9%), followed by wound-associated specimens (21.4%), blood (17.3%), respiratory samples (12.9%), and other sterile sites (4.8%).

**Figure 1.**
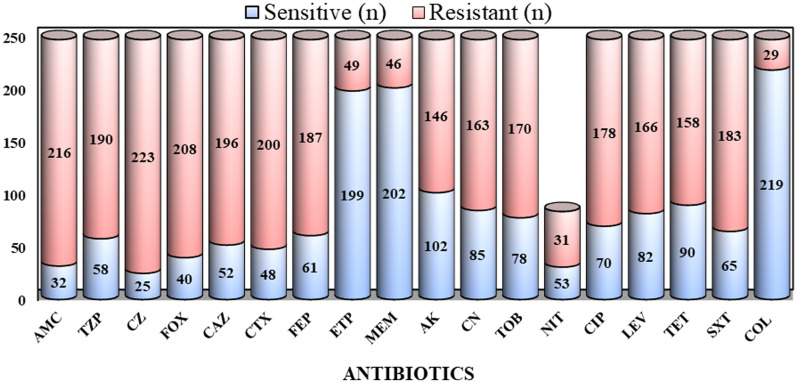
Antimicrobial susceptibility profile of *Klebsiella pneumoniae* isolates across tested antibiotic classes (n = 248). Amoxicillin–clavulanic acid (AMC), Piperacillin–tazobactam (TZP), Cefazolin (CZ), Cefoxitin (FOX), Ceftazidime (CAZ), Cefotaxime (CTX), Cefepime (FEP), Ertapenem (ETP), Meropenem (MEM), Amikacin (AK), Gentamicin (CN), Tobramycin (TOB), Nitrofurantoin (NIT), Ciprofloxacin (CIP), Levofloxacin (LEV), Tetracycline (TET), Trimethoprim–sulfamethoxazole (SXT), Colistin (COL).* for urine isolates only.

Antimicrobial susceptibility testing demonstrated high resistance to β-lactams (≥76%), cephalosporins (≥75%), and fluoroquinolones (≥65%). Carbapenem resistance ranged from 18% to 20%. Colistin (88.7% susceptible) and nitrofurantoin (63.0% susceptible among urinary isolates) retained the greatest activity (Figure 1). Overall, 60.5% of isolates were classified as MDR/XDR.

**Figure 2.**
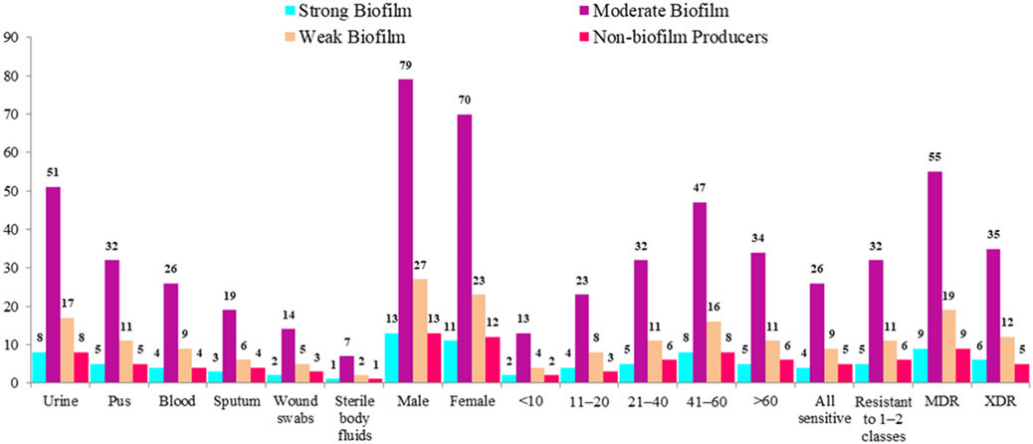
Distribution of biofilm-forming capacity among clinical isolates stratified by specimen type, demographic characteristics, and antimicrobial resistance profile (n = 248).

Hypermucoviscosity was observed in 41.5% of isolates. Biofilm formation was highly prevalent, with 89.9% of isolates demonstrating measurable biofilm production, predominantly of moderate intensity (Figure 2). These findings represent descriptive phenotypic frequencies only, as no molecular characterization or clonal analysis was performed.

## Discussion

This study provides a concise phenotypic characterization of antimicrobial resistance and selected virulence-associated traits among *K. pneumoniae* isolates in a high-burden healthcare setting. The high prevalence of MDR/XDR isolates and resistance to multiple antibiotic classes is consistent with regional and global trends and reflects sustained antimicrobial selection pressure in hospital environments.^8–10^

The retained activity of colistin and nitrofurantoin suggests their continued utility as last-line agents, particularly for urinary infections; however, concerns regarding toxicity and emerging resistance underscore the need for cautious use within antimicrobial stewardship frameworks.^8,9^

Biofilm formation was observed in the majority of isolates, consistent with its recognized role in persistence on medical devices and contribution to chronic and recurrent infections.^7–9^ Hypermucoviscosity was also frequently detected, although in the absence of molecular confirmation, this phenotype should be interpreted cautiously as a surrogate marker rather than definitive evidence of hypervirulence.^10^

Importantly, this study reports descriptive frequencies only, and no causal or statistical associations between antimicrobial resistance and virulence phenotypes can be inferred. While these findings are broadly consistent with existing literature, they provide region-specific data from a setting where integrated phenotypic surveillance remains limited. The relatively high proportion of *K. pneumoniae* among Gram-negative isolates warrants careful interpretation, as it may reflect local epidemiology, diagnostic practices, or patient population characteristics rather than broader trends.

Several limitations should be acknowledged. The absence of whole-genome sequencing precludes identification of resistance genes, virulence determinants, and mobile genetic elements. Similarly, lack of molecular typing limits inference regarding clonal relatedness and transmission dynamics across healthcare facilities. Additionally, the study design does not allow assessment of incidence or clinical outcomes, and findings reflect laboratory-based isolate frequencies rather than population-level burden.

## Conclusion

This study highlights a high burden of multidrug-resistant *Klebsiella pneumoniae* with frequent biofilm formation and hypermucoviscosity in tertiary-care hospitals. These findings underscore important therapeutic and infection control challenges. Strengthened surveillance and antimicrobial stewardship are needed, alongside molecular studies to better understand resistance mechanisms and transmission dynamics.

## Data Availability

The data supporting the findings of this study are available within the article.

## Final Approval of Manuscript

All authors reviewed and approved the final version of the manuscript and agreed to be accountable for all aspects of the work.
